# Sclerotic Acid

**Published:** 1878-02

**Authors:** 


### Sclerotic Acid.

This is the last therapeutic novelty. It is said to be the active principle of ergot, and has been isolated by Dragendorff. As yet its price is high, say $20 per ounce; but the dose is small, gr. $\frac{1}{12}$–$\frac{1}{16}$, by hypodermic injection.—*Med. and Surg. Reporter.*

